# Development of a Multiplex Bead Assay to Detect Serological Responses to *Brucella* Species in Domestic Pigs and Wild Boar with the Potential to Overcome Cross-Reactivity with *Yersinia enterocolitica* O:9

**DOI:** 10.3390/microorganisms10071362

**Published:** 2022-07-06

**Authors:** Antonia Touloudi, John McGiven, Shaun Cawthraw, George Valiakos, Polychronis Kostoulas, Lucy Duncombe, Christian Gortázar, Mariana Boadella, Marina Sofia, Zoi Athanasakopoulou, Dimitris C. Chatzopoulos, Vassiliki Spyrou, Liljana Petrovska, Charalambos Billinis

**Affiliations:** 1 Faculty of Veterinary Science, University of Thessaly, 431 00 Karditsa, Greece; atoul@uth.gr (A.T.); georgevaliakos@uth.gr (G.V.); msofia@uth.gr (M.S.); zathanas@uth.gr (Z.A.); 2 Department of Bacteriology, Animal and Plant Health Agency, OIE/FAO Brucellosis Reference Laboratory, Woodham Lane, Addlestone, Surrey KT15 3NB, UK; john.mcgiven@apha.gov.uk (J.M.); shaun.cawthraw@apha.gov.uk (S.C.); lucy.duncombe@apha.gov.uk (L.D.); 3 Faculty of Public and One Health, University of Thessaly, 431 00 Karditsa, Greece; pkost@uth.gr (P.K.); dchatzopoulos@uth.gr (D.C.C.); 4 SaBio, Instituto de Investigación en Recursos Cinegéticos IREC, 13005 Ciudad Real, Spain; christian.gortazar@uclm.es; 5 SABIOTEC, Camino de Moledores s/n, 13005 Ciudad Real, Spain; mariana.boadella@gmail.com; 6 Faculty of Animal Science, University of Thessaly, 412 22 Larissa, Greece; vasilikispyrou@uth.gr

**Keywords:** multiplex bead assay, *Brucella suis*, wild boar, domestic pigs, cross-reactivity, rLPS antigen, smooth *Brucella suis* 1330 antigen, *Yersinia enterocolitica* O:9 antigen

## Abstract

The aim of this study was to develop a multiplex bead assay using a *Brucella* rLPS antigen, a *Brucella suis* smooth antigen, and a *Yersinia enterocolitica* O:9 antigen that not only discriminates *Brucella*-infected from *Brucella*-uninfected pigs and wild boar, but also overcomes the cross reactivity with *Y. enterocolitica* O:9. Sera from 126 domestic pigs were tested: 29 pigs were *Brucella* infected, 80 were non-infected and 17 were confirmed to be false positive serological reactors (FPSR). Sera from 49 wild boar were tested: 18 were positive and 31 were negative. Using the rLPS antigen, 26/29 *Brucella*-infected domestic pigs and 15/18 seropositive wild boar were positive, while 75/80 non-*Brucella* infected domestic pigs, all FPSR, and all seronegative wild boar were negative. Using the smooth *B. suis* 1330 antigen, all *Brucella*-infected domestic pigs, 9/17 FPSR and all seropositive wild boar were positive, while all non-infected pigs and 30/31 seronegative wild boar were negative. The ratio of the readouts from the smooth *B. suis* antigen and *Y. enterocolitica* O:9 antigen enabled discriminating all *Brucella* infected individuals from the FPSR domestic pigs. These results demonstrate the potential of this assay for use in the surveillance of brucellosis, overcoming the cross-reactivity with *Y. enterocolitica*.

## 1. Introduction

Porcine brucellosis is a major concern and is widespread throughout the world, especially in the Mediterranean, Balkans, South America and South East Asia. The primary etiologic agent is the bacterium *Brucella suis*, and the disease is a cause of severe economic losses in livestock production and may threaten public health [1]. The Eurasian wild boar (Sus scrofa) is widely distributed in the Palearctic and is a wildlife reservoir host for *B. suis* in many regions. The apparent prevalence of *B. suis* (based on serology) has been estimated to range from 25–46% in areas of high wild boar density in Spain where epidemiological links with *Brucella* infection in domestic pigs are suspected [2,3].

Serological methods used for diagnosis of porcine brucellosis include indirect, blocking and competitive enzyme-linked immunosorbent assays (ELISA) based on smooth lipopolysaccharide antigens (sLPS), the Rose Bengal Test (RBT), the complement fixation test (CFT) and the fluorescence polarization assay [1,4]. The *B. abortus* antigens seem to be suitable for testing swine sera, at least in RBT and CFT, as they can identify antibodies against the three biovars (1,2,3) of *B. suis*, which infect pigs [1]. A drawback of these serological tests is the lack of reliability for individual diagnosis, because, although they may have acceptable sensitivity, they frequently lack specificity [1]. A major reason for this is infection by *Yersinia enterocolitica* O:9, which has antigenic determinants (sLPS O-chains) closely related to those of *Brucella* spp. [5,6,7,8,9]. The structure and the biological properties of the rough *Brucella* LPS make it a suitable antigen for the serodiagnosis of porcine brucellosis [10]. Specifically, it lacks O-chain and only the lipid A and the core antigens remain. This rLPS structure differs between *Brucella* and *Y. enterocolitica* O:9 [11,12,13]. The omission of the cross-reactive O-chain means that rLPS has the potential to be a more specific antigen when applied to samples that are false-positive in assays employing the O-chain [14]. In wild boar, the sensitivity of a multi-species *Brucella* sLPS iELISA was estimated at being 100% and its specificity was adequate. However, its cross-reactivity with *Y. enterocolitica* O:9 was not assessed [2].

The above reasons prompted us to use an rLPS-rich antigen, extracted from a rough strain of *Brucella*, in order to try to enhance the specificity (Sp) of serology. Additionally, a whole cell *B. suis* biovar 1 (strain 1330) smooth antigen was used to maximize sensitivity (Se) because it is a homologous antigen for infected domestic pigs and wild boar. Being a whole-cell preparation, it contains the most possible immunogenic epitopes, and has a high O-chain content, which includes a low frequency of the OPS M epitope that is not possessed by *Y. enterocolitica* O:9 [1,15]. The combination of these two antigens in the same multiplex bead assay could enhance the diagnostic accuracy. Finally, a whole cell *Y. enterocolitica* O:9 antigen was used to detect cross-reacting antibodies and/or antibodies produced after natural exposure to *Y. enterocolitica* that may be present in *Brucella*-seropositive domestic pigs and wild boar.

The purpose of the present study was to develop a multiplex bead assay using *Brucella* rLPS, a whole cell *B. suis* 1330 smooth, and a whole cell *Y. enterocolitica* O:9 antigen, which not only discriminates *Brucella*-seropositive from *Brucella*-seronegative domestic pigs and wild boar but also overcomes the cross reactivity between *B. suis* and *Y. enterocolitica* O:9.

## 2. Materials and Methods

### 2.1. Domestic Pig Sera

One-hundred and twenty-six domestic pig sera were used for test development. Group A contained 29 sera from animals that were culture-positive for *B. suis* biovar 2 (25/29, obtained in Spain), or biovar 1 (4/29, obtained in South America). Group B contained 80 randomly selected sera collected from herds within Great Britain, which is officially brucellosis-free. Group C contained 17 sera from herds within Great Britain that were FPSR (false-positive serological reactors) during routine testing by either RBT (*n* = 10), cELISA (*n* = 10), SAT (*n* = 8) or their combination.

### 2.2. Wild Boar Sera

Sera from 49 Eurasian wild boar from Spain were also tested: group A—*Brucella* seropositive, (*n* = 18)—and group B—*Brucella* seronegative (*n* = 31). The discrimination between the seropositive and seronegative wild boars was determined by an indirect ELISA using the sLPS antigen [2].

### 2.3. Multiplex Bead Assay

#### 2.3.1. Antigen Preparation and Coupling

The antigens used for assay development: (a) rLPS-rich phenol/chloroform/petroleum ether extract from *B. abortus* RB51 (hereafter referred as rLPS) [14]; (b) whole cells of the smooth *B. suis* strain 1330 grown on serum dextrose agar at 37 °C and heat-killed [16]; (c) whole cells of the smooth *Y. enterocolitica* O:9 (strain 234/02) grown on nutrient agar at 27 °C and heat-killed. Ten micrograms of each antigen were bound to 2.5 × 10^6^ Pro Magnetic carboxylated beads according to the manufacturer’s instructions (Bioplex Pro Magnetic COOH Magnetic Beads Amine Coupling Kit, BioRad, Hercules, CA, USA).

#### 2.3.2. Multiplex Bead Assay Protocol

The Bio-Rad Bio-Plex multi-analyte bead suspension array system, which is based on Luminex’s xMAP technology, was used for the assay. The bead reporter fluorescence, expressed as MFI (median fluorescence intensity), was determined with a Bio-Plex 200 (Bio-Rad) instrument that was initially calibrated and set to count 100 beads from each of three bead sets, with the Double Discriminator (DD) gate values set at 7500–25,000. A one-step protocol and normalization method for MFI values were used, as described previously [17]. Fifty microliters of master mix, containing approximately 3500 coupled beads of each type (the rLPS antigen, the smooth *B.suis* 1330 antigen and the smooth *Y. enterocolitica* O:9 antigen), biotinylated protein AG (secondary antibody) at a 1:500 dilution and streptavidin–phycoerythrin (2 μg/mL) in dilution buffer, were added to each well of a flat-bottom 96-well plate. Diluted serum (50 μL) was mixed with the master mix and the plate was incubated for 2 h at room temperature, with shaking at 600 rpm. The beads were washed twice with 100 μL Wash buffer (0.1 M PBS and 0.05% Tween 20) using the Bioplex pro Wash Station (BioRad) and finally resuspended in 100 μL of dilution buffer. The bead reporter fluorescence, expressed as MFI, was determined using Bioplex 200 (BioRad) instrument. The machine was calibrated and set to count 100 beads from each of three bead sets. Serum from a known seropositive *Brucella*-infected domestic pig was included as a positive control on each plate for normalization of test sera MFI values.

### 2.4. Receiver Operating Characteristic (ROC) Curve Analysis

ROC analysis was used for (i) evaluating overall test performance and (ii) determining cut-points that optimize the diagnostic accuracy of the test. For (i) the trapezoidal rule [18] was used to calculate the area under the ROC curve (AUC) and the corresponding confidence intervals [19]. For (ii) two different criteria were used for cut-point selection: (a) the simultaneous optimization of the Se and Sp and the overall minimization of false results, which corresponds to the maximization of the Youden’s index J = Se + Sp − 1 [20] and (b) the minimization of the quantity min (1 − Se)^2^ + (1 − Sp)^2^ that corresponds to the cut-point closest to the upper left corner of the AUC plot [21].

ROC analysis was performed to compare the following combinations: (I) group A and B for the rLPS antigen in domestic pigs; (II) group A and C for the rLPS antigen in domestic pigs; (III) group A and B for the *B. suis* 1330 smooth antigen in domestic pigs; (IV) group A and C for the *B. suis* 1330 smooth antigen in domestic pigs; (V) group A and C for the ratio of the readout from the smooth *B. suis* 1330/the readout from the smooth *Y. enterocolitica* O:9 in domestic pigs; (VI) group A and B for the rLPS antigen in wild boars; (VII) group A and B for the *B. suis* 1330 smooth antigen in wild boars.

All analyses were carried out in R [22], using the pROC package [23].

## 3. Results

**Figure 1:**
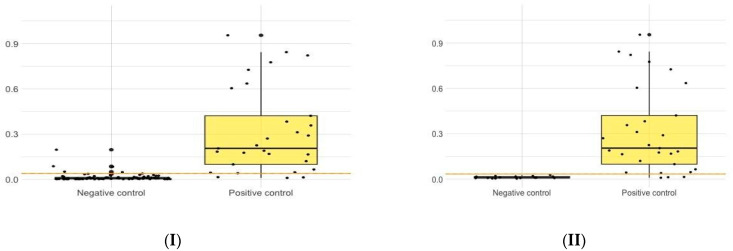

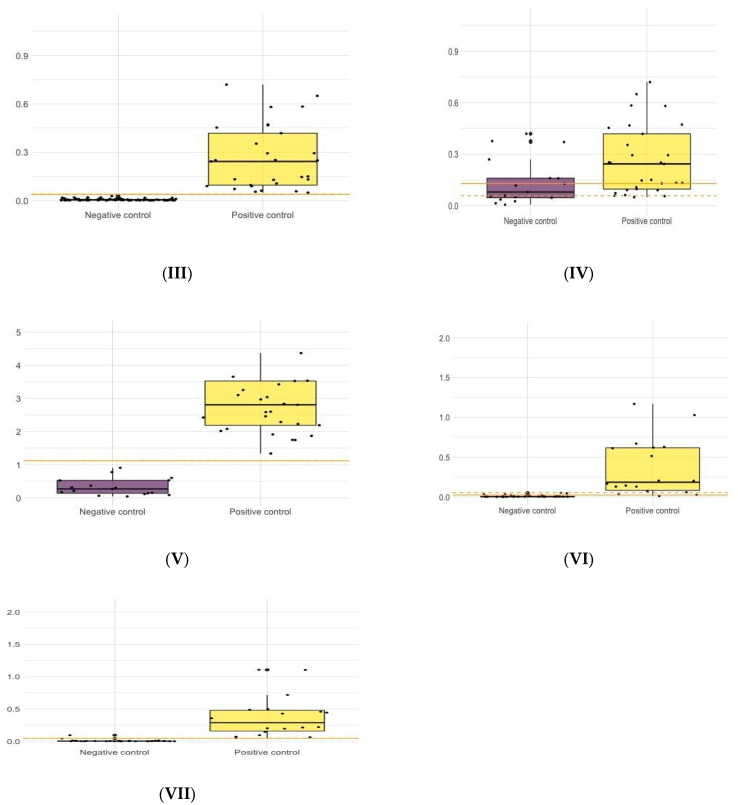
Distribution of the test values for the following combinations: group A and B for the rLPS antigen in domestic pigs (**I**); group A and C for the rLPS antigen in domestic pigs (**II**); group A and B for the smooth *B. suis* 1330 antigen in domestic pigs (**III**); group A and C for the smooth *B. suis* 1330 antigen in domestic pigs (**IV**); group A and C for the ratio of smooth *B. suis* 1330/the smooth *Y. enterocolitica* O:9 in domestic pigs (**V**); group A and B for the rLPS antigen in wild boars (**VI**); group A and B for the smooth *B. suis* 1330 antigen in wild boars (**VII**). Dots correspond to actual values. Boxes represent interquartile ranges while the solid black line at the approximate center of each box is the median; the arms of each box extend to cover the central 95% of the distribution of the normalized values.

**Figure 2:**
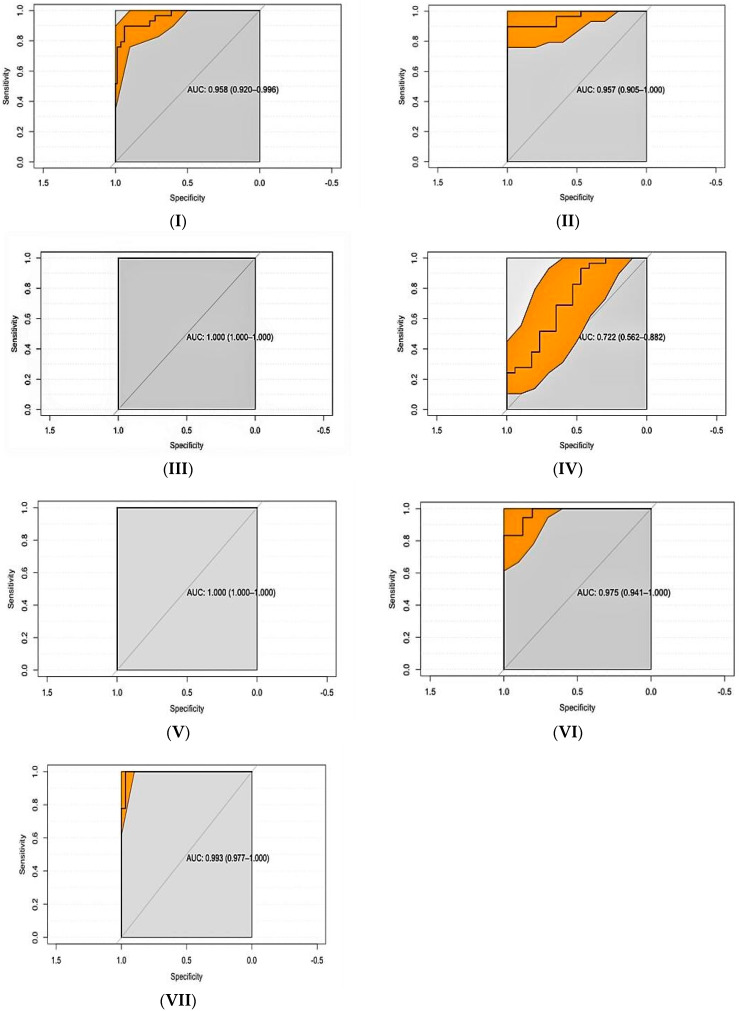
Plot of the Receiver Operating Characteristic (ROC) curve analysis for the following combinations: group A and B for the rLPS antigen in domestic pigs (**I**); group A and C for the rLPS antigen in domestic pigs (**II**); group A and B for the smooth *B. suis* 1330 antigen in domestic pigs (**III**); group A and C for the smooth *B. suis* 1330 antigen in domestic pigs (**IV**); group A and C for the ratio of smooth *B. suis* 1330/the smooth *Y. enterocolitica* O:9 in domestic pigs (**V**); group A and B for the rLPS antigen in wild boars (**VI**); group A and B for the smooth *B. suis* 1330 antigen in wild boars (**VII**).

The distribution of the normalized values for each species are shown in Figure 1. ROC curves are shown in Figure 2. The overall discriminatory power for all combinations was high as indicated by the AUCs, which were, in all instances, higher than 0.95 except for the smooth antigen between groups A and C in domestic pigs which was 0.722 (Table 1). Se and Sp combinations at the selected cut-offs were also high for all combinations with the exception of the *B. suis* 1330 smooth antigen between groups A and C in domestic pigs.

The selected cut-offs that maximize the Youden’s J statistic showed that 26/29 group A domestic pigs (23/25 *Brucella* infected by biovar 2 and 3/4 infected by biovar 1) were positive using the rough *B. abortus* RB51 antigen, while 75/80 group B domestic pigs and all (17/17) group C (FPSR) domestic pigs were negative. The same antigen detected 15/18 of the seropositive wild boar and was negative for all (31/31) seronegative wild boar. The smooth *B. suis* 1330 antigen detected in all (29/29) group A domestic pigs was negative in all (80/80) group B animals, but was positive in just over half (9/17) of the group C (FPSR) samples. In wild boars, the same antigen was detected in all (18/18) group A seropositive samples and was negative in 30/31 group B seronegative animals. Finally, the ratio of the smooth *B. suis* 1330 and the smooth *Y. enterocolitica* O:9 normalized MFI values discriminated with 100% sensitivity and 100% specificity between group A and group C (FPSR) domestic pigs.

## 4. Discussion

This study shows that a multiplex bead assay could be a useful serodiagnostic tool for porcine brucellosis due to the high Se and Sp (whole cell *B. suis* 1330 smooth antigen) and the ability to identify cross-reactions due to *Y. enterocolitica* O:9 (rLPS antigen; smooth *B. suis* 1330/smooth *Y. enterocolitica* O:9 normalized MFI values ratio).

The most commonly used serological tests are generally designed to measure antibodies against a single antigen preparation, whereas the one-step multiplex bead assay is capable of detecting antibody responses to a range of different antigens at the same time. This offers significant benefits over other tests, including reduced reagent costs [24]. The potential advantages of multiplex bead assays over conventional serologic tests provide a strong impetus for their routine use in both research and clinical laboratories. In our study the beads were conjugated on three separate occasions, one conjugation per antigen and bead type, and were easily mixed and efficiently combined.

The frequency of the false-positive reactions in group B domestic pigs and wild boar was low (<10%), despite the fact that multiplexed assays are usually characterized by a lower Sp when compared to conventional serological tests due to the simultaneous presence of multiple ligands [25]. One possible explanation could be the use of protein AG instead of a species/isotype-specific secondary antibody. The results of this study show that the multiplex bead assay using rLPS and smooth *B. suis* 1330 antigens effectively distinguishes between sera from group A and group B in both domestic pigs and wild boar. Based on the calculated AUC values, Se and Sp of the latter antigen seems to be better than rLPS for this purpose.

In a recent study, the Se and Sp of a conventional iELISA, in which the sLPS antigen was used, were 0.66 and 0.97, respectively, leading to the conclusion that this assay is not sensitive enough for the diagnosis of brucellosis in domestic pigs [26]. Furthermore, in another study the use of sLPS in an iELISA showed a DSn of 95.07% and a DSp of 99.75% but only 24.77 with FPSR samples, results similar to ours [27]. In another study, the use of sLPS antigen in the iELISA resulted in 0.94 Se and 1.00 Sp [14], confirming the results of several previous publications that also support the diagnostic accuracy of sLPS in discriminating *Brucella*-infected from non-infected domestic pigs [28,29]. Our results indicate that a multiplex bead assay using the whole smooth *B. suis* 1330 antigen may be better than conventional serologic tests at discriminating between sera from *Brucella* infected and non-infected non-FPSR domestic pigs (Se: 1.00, Sp: 1.00).

The use of rLPS had a satisfactory diagnostic performance in the multiplex bead assay. The good distinction between group A and group B samples from domestic pigs was also found in a recent study where the same antigen was used in an iELISA, resulting in a Se of 0.91 and a Sp of 0.99 [14]. The results of the multiplex bead assay clearly show that in domestic pigs, the rLPS antigen can discriminate group A (*Brucella* infected animals) from group C (non-*Brucella* infected FPSR) sera. This attribute of the rLPS rich antigen was anticipated, as the structure of the core sugars within the rLPS is very different between *Brucella* spp. [30] and *Y. enterocolitica* O:9 [13] and the absence of the O-chain in this antigen [31] avoids cross-reactivity with antibodies against *Y. enterocolitica* O:9, as has been previously shown using an iELISA method [14]. Other studies have shown *Brucella* rLPS antigen to be less effective at serodiagnosis [27]. However, in this case, the antigen was pure and so differs from the less pure preparation used in this study, within which the co-extractants may behave as excipients and enhance the efficacy of the rLPS antigen.

The application of serological tests in wildlife is usually carried out for screening purposes or surveillance. Wild boar are indigenous in many countries and may contribute to the transmission of *B. suis* to livestock and hamper the success of eradication programs [32]. Based on our results, the best antigen for screening wild boar populations with the multiplex bead assay is the smooth *B. suis* 1330 (Se: 1.00, Sp: 0.97). Furthermore, given the high seroprevalence (up to 63%) against *Brucella* spp. in European wild boar populations [33], the concomitant use of rLPS may improve the combined specificity, considering that this antigen gave a negative result in only one group B serum sample, with a positive normalized MFI value for the smooth *B. suis* 1330 antigen. Further studies with larger sample sizes are obviously needed to confirm this hypothesis.

According to a previous study [34], the cut-off should be selected by taking into consideration the epidemiologic situation in each area. For example, for countries which are brucellosis-free, by taking into account the low prevalence of the disease and the serious consequences of a false-positive diagnosis, it may be advisable to choose a cut-off at the lower part of the ROC curve in order to maximize the Sp. On the other hand, a maximum Se would be appropriate for countries where the disease occurs at high prevalence. Therefore, the Se and Sp of the multiplex bead assay may change depending on the criteria used for cut-off selection.

The poor ability of the smooth *B. suis* 1330 antigen to differentiate between group A and group C domestic pigs was also expected based on previous studies in cattle [35,36] and pigs [8,9], as the antigen did differentiate between most of the samples, but not nearly so well as the rLPS antigen, However, the concomitant use of the smooth *Y. enterocolitica* O:9 antigen in the multiplex bead assay and the calculation of the ratio between the smooth *B. suis* 1330 and the smooth *Y. enterocolitica* O:9 normalized MFI values fully overcame this drawback, permitting the clear differentiation between groups A and C. This would most likely be due to the binding of antibodies on each antigen that are not shared but are distinct to each antigen types. Furthermore, the use of the rLPS antigen in a multiplex bead assay may be helpful in cases of dual infections of *Brucella* spp. and *Y. enterocolitica* O:9, given that the OPS is so similar between the *B. suis* biovar 2 and the *Y. enterocolitica* O:9 [15].

## 5. Conclusions

Based on the results of this study, the multiplex bead assay can be considered to be an accurate diagnostic test for brucellosis in domestic pigs and wild boar, if at least two antigens are included. For domestic pigs, the use of the smooth *B. suis* 1330 antigen along with the *Y. enterocolitica* O:9 antigen (thus enabling calculation of the ratio between MFI values for the two antigens) seems to be the best combination to discriminate between sera from *Brucella*-infected and non-*Brucella*-infected (FPSR and non-FPSR) animals, although the addition of the rLPS would help in the case of dual infection. In wild boar, the smooth *B. suis* 1330 antigen seems to be more accurate in terms of Se and Sp but the addition of the rLPS may further increase Sp.

## Figures and Tables

**Table 1:** Estimated AUCs, Se, Sp and associated 95% Confidence Intervals (CIs) at selected cut-offs that maximize the Youden’s J statistic (1) or correspond to the point closest to upper left corner of the AUC plot (2). Results are for the following combinations: group A and B for the rLPS antigen in domestic pigs (I); group A and C for the rLPS antigen in domestic pigs (II); group A and B for the *B. suis* 1330 smooth antigen in domestic pigs (III); group A and C for the *B. suis* 1330 smooth antigen in domestic pigs (IV); group A and C for the ratio of smooth *B. suis* 1330/the smooth *Y. enterocolitica* O:9 in domestic pigs (V); group A and B for the rLPS antigen in wild boars (VI); group A and B for the *B. suis* 1330 smooth antigen in wild boars (VII).

Combination	AUC (CIs)	Cut-Off (1)			Cut-Off (2)		
			Se (CIs)	Sp (CIs)		Se (CIs)	Sp (CIs)
**I**	0.958 (0.920; 0.996)	0.039	0.90 (0.79; 1.00)	0.94 (0.88; 0.98)	0.039	0.90 (0.79; 1.00)	0.94 (0.88; 0.98)
**II**	0.957 (0.905; 1.000)	0.034	0.89 (0.76; 1.00)	1.00 (1.00; 1.00)	0.034	0.89 (0.76; 1.00)	1.00 (1.00; 1.00)
**III**	1.000 (1.000; 1.000)	0.039	1.00 (1.00; 1.00)	1.00 (1.00; 1.00)	0.039	1.00 (1.00; 1.00)	1.00 (1.00; 1.00)
**IV**	0.722 (0.562; 0.882)	0.058	0.93 (0.83; 1.00)	0.47 (0.23; 0.71)	0.128	0.69 (0.52; 0.83)	0.65 (0.41; 0.88)
**V**	1.000 (1.000; 1.000)	1.123	1.00 (1.00; 1.00)	1.00 (1.00; 1.00)	1.123	1.00 (1.00; 1.00)	1.00 (1.00; 1.00)
**VI**	0.975 (0.941; 1.000)	0.056	0.83 (0.67; 1.00)	1.00 (1.00; 1.00)	0.026	0.94 (0.83; 1.00)	0.87 (0.74; 0.97)
**VII**	0.993 (0.977; 1.000)	0.047	1.00 (1.00; 1.00)	0.97 (0.90; 1.00)	0.047	1.00 (1.00; 1.00)	0.97 (0.90; 1.00)

## Data Availability

All data are presented in the manuscript.
